# Perpetrator and situational characteristics associated with security alerts in regional Australian emergency departments

**DOI:** 10.1186/s12873-022-00608-6

**Published:** 2022-03-24

**Authors:** Brodie Thomas, Peter O’Meara, Kristina Edvardsson, Damhnat McCann, Evelien Spelten

**Affiliations:** 1 La Trobe Rural Health School, La Trobe University, Mildura, Australia; 2 Department of Paramedicine, Monash University, Frankston, Australia; 3 School of Nursing and Midwifery, La Trobe University, Bundoora, Australia; 4 Judith Lumley Centre, La Trobe University, Melbourne, Australia; 5 School of Nursing, University of Tasmania, Launceston, Australia

**Keywords:** Workplace violence, Emergency service, Hospital, Risk management, Occupational injuries, Security alert, Code black

## Abstract

**Background:**

Workplace violence is a regular feature of emergency departments (ED) and reported to be increasing in frequency and severity. There is a paucity of data from regional EDs in Australia. The aim of this study was to identify the perpetrator and situational characteristics associated with security alerts in regional emergency departments.

**Methods:**

This retrospective descriptive study was conducted in two regional Australian hospital EDs. All incident reports, hospital summary spreadsheets, and patient medical records associated with a security alert over a two-year period (2017 - 2019) were included. The situational and perpetrator characteristics associated with security alerts in the ED were recorded.

**Results:**

One hundred fifty-one incidents were reported in the two-year period. Incidents most frequently occurred on late shifts and in an ED cubicle. Most incidents included multiple disciplines such as ED staff and paramedics, police and psychiatric services. One hundred twenty-five incidents had sufficient information to categorise the perpetrators. Mental and behavioural disorders (MBD) were the most frequent perpetrator characteristic present in security alerts (*n* = 102, 81.6%) and were associated with increased severity of incidents. MBDs other than psychoactive substance use (PSU) were associated with 59.2% (*n* = 74) of incidents and 66.7% (*n* = 18) of injuries. PSU was associated with 42.4% (*n* = 53) of incidents. Following PSU and MBDs other than PSU, repeat perpetrators were the next most prominent perpetrator category (24.8% *n* = 31) and were almost always associated with an MBD (93.5% *n* = 29).

**Conclusions:**

Violence incidents in the ED are often complex, patients present with multiple issues and are managed across disciplines. Interventions need to extend from one size fits all approaches to targeting specific perpetrator groups. Since MBDs are one of the most significant perpetrator factors, interventions focussing on this characteristic are needed to address workplace violence in EDs.

## Background

Emergency departments (EDs) can be chaotic, stressful and high-risk environments for staff [1, 2]. Workplace violence is a regular feature of this environment and reported to be increasing in frequency and severity [1, 3, 4]. The consequences of workplace violence include negative individual physical and psychological impacts, as well as organisational and societal effects [4–7].

Most interventions to reduce workplace violence focus on education and training for staff as a one-size fits all approach [2, 8]. However, there is very weak evidence of their effectiveness, suggesting interventions should be more targeted in their design. Including a focus on perpetrators may provide a more informed and tailored approach to violence prevention [2, 6, 7, 9]. In previous literature, perpetrators are generally categorised into patients, family members or others [10]. Emergency department nurses in Australia have indicated that they differentiate between violent indivduals, placing them among six categories. They then tailor their approach based on which category they fall in to. The six categories of perpetrators are: (i) No medical cause, (ii) Mental Health, (iii) Physical Health, (iv) Substance abuse/addiction, (v) Complexity of issues, (vi) Repeat perpetrators [2].

While qualitative descriptions have categorised perpetrators of violence based on the apparent underlying cause for aggression [2], the epidemiology of violence in the ED is not clear due to a lack of empirical data [3, 9]. The majority of studies investigating characteristics of workplace violence use staff surveys, focussing on staff perception rather than objective incident data [1, 10]. Survey data has indicated that alcohol, drug intoxication and mental health issues are the most prevalent factors associated with violence in EDs, however the subjective self-reports from staff do not paint the whole picture [9, 11]. Recent reviews of the international literature highlight several studies, spanning over 25 years, that report on characteristics of violent incidents in EDs, and indicate that few studies include data sources beyond incident reports [1, 12]. Only four studies have incorporated incident reports and patient medical notes to provide detailed understanding of the characteristics of perpetrators. Each of these used varying methods and objectives, and all were conducted in metropolitan EDs [1, 12]. The consistent findings from hospital records indicate that verbal violence is most common type of violence, [1, 3, 10, 13], perpetrators are predominantly male [1, 9, 14–16], and psychoactive substance use is the most common precipitant to violence [1, 3, 9, 12, 14–17]. Mental health issues as precipitants to violence is commonly yet inconclusively reported, with rates of associated mental health issues ranging from 14 to 78% [1, 9, 12, 15].

Two audits of incident reports have been conducted in regional Australian EDs, one over a five-year period and one over a six-month period [3, 16]. The studies reported increasing rates of violent events at comparable levels to metropolitan EDs with higher severity. Both studies found males to perpetrate violence more commonly than females and both reported high rates of psychoactive substance use. Mental health issues associated with violence were reported in one quarter of cases in one study and not reported in the other [3, 16]. Further data from regional and rural EDs in Australia are needed to complement the existing literature [1, 12] and studies reporting the influencing factors behind violence are required to provide a better understanding of the breadth and depth of the problem and more targeted efforts to reduce violence [10, 13].

### Study aim

The aim of this study was to identify the perpetrator and situational characteristics associated with security alerts in the emergency department to inform future planning for targeted and evidence-based interventions to prevent workplace violence in EDs.

## Methods

### Study design

This is a retrospective descriptive study of incident reports, hospital summary spreadsheets, and identified patient medical records associated with a security alert in EDs over a two-year period.

### Study setting

This study was conducted at two regional Victorian sites: a 724 bed (level 2) tertiary teaching hospital in inner regional Victoria and a 165 bed (level 1) tertiary teaching hospital in outer regional Victoria. In this Australian State, security alerts are either Code Black or Code Grey; Code black is a police and security response to an armed threat while a Code Grey is an organisational-wide clinical and security response to actual or perceived violence or aggression [3, 18].

### Data collection

Data were collected between 26 January 2017 and 25 January 2019 using an adapted version of an audit tool developed and piloted in a regional Tasmanian hospital (e-mail from Dr. Damhnat McCann, April 2017). Summary spreadsheets were provided as Microsoft excel spreadsheets, incidents reports were accessed via Victorian Health Incident Management System, and patient records were accessed electronically for site 1 and via physical records at site 2.

### Data analysis

Data were collected via the audit tool and analysed in SPSS [19]. Descriptive statistics were used for the individual and situational variables associated with a security alert in the ED. Missing data were treated as missing completely at random.

The categories of perpetrators were pre-determined using the categories highlighted by ED nurses with slight modifications to align with the WHO International Classification of Disease and Health Problems which are utilised in the EDs (WHO ICD) [2, 20]. The main modification is that the WHO ICD considers substance abuse and addiction to be a mental or behavioural disorder, therefore psychoactive substance use, other mental and behavioural disorders and complex mental and behavioural disorders have been included as subcategories of a single, more inclusive category, mental and behavioural disorders [20]. The categories include violence associated with (i) no medical problem (e.g. frustrated with waiting), (ii) physical health issue, (iii) mental and behavioural disorders (MBD), and (iv) repeat perpetrators. The MBD category has three sub-categories: Psychoactive substance abuse (PSU), MBDs other than PSU, and complex MBDs [2, 20]. Perpetrators were placed into the pre-defined categories based on patient presenting problems, diagnoses and narrative accounts in incident reports and patient notes. Previous medical history was collected from previous diagnoses that were recorded in the patient medical records, it is not known if these diagnoses were made in the ED or in other contexts. Perpetrators were considered repeat perpetrators if they were responsible for security alerts over multiple presentations or if an alert for previous violent behaviour was recorded. Those responsible for multiple security alerts during a single presentation were not considered repeat perpetrators.

Violence is a complex phenomenon and there may be many contributing factors. Because of this, perpetrators cannot be assumed to have only one associated contributing factor and may fit into multiple categories. The categories of perpetrators are not mutually exclusive which results in non-mutually exclusive nominal variables. Two other characteristics contained non-mutually exclusive variables; the nature of the incident and MBD past history. Placing them into mutually exclusive categories was not practical as it would result in an excessive number of individual variables with low numbers. As this study reports descriptive data, non-mutually exclusive categories did not negatively impact on the results and further adjustment was not necessary. All characteristics including demographic information is reported based on the incidents, not on individual perpetrators. For example, the sex of the perpetrator will be reported for the 127 incidents as opposed to the 108 individual perpetrators where sex was identified.

### Ethics approval

Ethics approval was obtained from the Bendigo Health Human Research Ethics Committee and covered both sites (HREC Reference Number: LNR/17/BHCG/55). All methods were carried out in accordance with relevant guidelines and regulations. Written consent to access data was provided by the Director of the ED at each Hospital. The requirement for informed consent to access individuals’ medical records was waived by the Bendigo Health Human Research Ethics Committee.

## Results

The audit of summary spreadsheets identified 151 security alert incidents over the two-year period. Incident reports were not created for 26 incidents. A total of 125 incident reports and 96 individual patient medical records were audited. Fourteen perpetrators had multiple incidents recorded in their medical records resulting in 114 incidents being recorded in medical records. Table 1 presents the incidence of security alerts at each health service over the two-year period.

**Table 1:** Incidence of security alerts

Location	Time period		
	26/1/2017-25/1/2018 n	26/1/2018-25/1/2019 n	Total study period n
Health service 1			
Security alerts	17	27	44
Patient presentations	52,088	54,141	106,229
Security alerts per 1000 presentations	0.33	0.50	0.41
Health service 2			
Security alerts	38	69	107
Patient presentations	33,436	34,255	67,691
Security alerts per 1000 presentations	1.14	2.01	1.58
Combined			
Security alerts	55	96	151
Patient presentations	85,524	88,369	173,920
Security alerts per 1000 presentations	0.64	1.09	0.87

### Perpetrator and situational characteristics

Perpetrator characteristics are presented in Table 2 and situational characteristics in Table 3. Most variables had missing data, the totals in Tables 2 and 3 have been adjusted to reflect the data considered to be missing completely at random.

**Table 2:** Perpetrator characteristics

Perpetrator characteristics of 151^**a**^ incidents			
	n (%)		n (%)
**Age (*n*** **= 123)**		**MBD past history (*n*** **= 125)**^**b**^	
Adolescent < 15	1 (0.8)	Previous psychiatric diagnosis	87 (69.6)
Young adult 15-34	55 (44.7)	- *Depression*	39 (31.2)
Middle aged 35-54	47 (38.2)	- *Bipolar*	20 (16.0)
Older adult > 54	20 (16.3)	- *Schizophrenia*	20 (16.0)
**Sex (*n*** **= 127)**		- *Anxiety*	16 (12.8)
Male	79 (61.9)	- *Dementia*	7 (5.6)
Female	48 (38.1)	- *Psychosis*	6 (4.8)
**Perpetrator category (*n*** **= 125)**^**b**^		- *Intellectual disability*	6 (4.8)
No medical cause	18 (14.4)	- *Autism spectrum disorder*	4 (3.2)
Physical health	11 (8.8)	- *Other*	31 (24.8)
Mental and behavioural disorder (MBD)	102 (81.6)	- *Multiple conditions*	47 (37.6)
- *Psychoactive substance use (PSU)*	53 (42.4)	**Alert for violence (*n*** **= 114)**	
- *MBD other than PSU*	74 (59.2)	Present in medical record	19 (16.7)
- *MBD Complex*	22 (17.6)	**Assessment order (*n*** **= 125)**	
Repeat perpetrators	31 (24.8)	Currently under assessment order	13 (10.4)
**Psychoactive substance use (*n*** **= 125)**		**Discharge (*n*** **= 88)**	
Any psychoactive substance use	53 (42.4)	Home	38 (43.2)
- *Drug use only*	26 (20.8)	Admitted to mental health unit	24 (27.3)
- *Alcohol use only*	19 (15.2)	Admitted to medical unit	17 (19.3)
- *Drug and alcohol use*	8 (6.4)	Other	9 (10.2)

^a^Characteristics with totals less than 151 represent missing data

^b^Categories are not mutually exclusive

**Table 3:** Situational characteristics

Situational characteristics of 151^**a**^ incidents			
	n (%)		n (%)
**Day of week (*n*** **= 151)**		**Triage category (*n*** **= 93)**	
Mon	21 (13.9)	Category 1: Immediate life threat	0 (0.0)
Tues	15 (9.9)	Category 2: Emergency	12 (12.9)
Wed	18 (11.9)	Category 3: Urgent	52 (55.9)
Thu	23 (15.2)	Category 4: Semi-urgent	25 (26.9)
Fri	25 (16.6)	Category 5: Non-urgent	4 (4.3)
Sat	24 (15.9)	**Time in ED prior to alert (*n*** **= 93)**	
Sun	25 (16.6)	< 60 min	24 (25.8)
**Shift type (*n*** **= 143)**		1-4 h	42 (45.2)
Early (0700-1530)	42 (29.4)	> 4 h	27 (29.0)
Late (1530-2200)	59 (41.3)	**Nature of incident (*n*** **= 133)**^**b**^	
Night (2200-0700)	42 (29.4)	Verbal violence	96 (72.2)
**Location of event (*n*** **= 114)**		Physical violence	70 (52.6)
Main ED cubicle	39 (34.2)	Absconding	28 (21.1)
Waiting room	22 (19.3)	Damage to property	18 (13.5)
Triage area	18 (15.8)	Self-harm	14 (10.5)
Resuscitation bay	8 (7.0)	**Weapon (*n*** **= 132)**	
Ambulance waiting area	7 (6.1)	In possession	21 (15.9)
Hallway	7 (6.1)	**Type of weapon (*n*** **= 21)**	
Other	13 (11.4)	Near object	13 (61.9)
**Type of perpetrator (*n*** **= 131)**		Other	8 (38.1)
Patient	123 (93.9)	**Injuries (*n*** **= 133)**	
Bystander	8 (6.1)	Incidents where Injury occurred	26 (19.5)
**Bystanders present (*n*** **= 127)**		**Injured person (*n*** **= 27)**^**c**^	
Yes	24 (18.9)	Staff	21 (77.8)
No	103 (81.1)	Patient	6 (22.2)
**Ambulance (*n*** **= 105)**		**Management (*n*** **= 128)**	
Arrival via Ambulance	64 (61.0)	Sedation only	29 (22.7)
**Police (*n*** **= 109)**		Restraint only	10 (7.8)
Police involvement	61 (56.0)	Sedation and restraint	23 (18.0)

^a^Characteristics with totals less than 151 represent missing data

^b^Categories are not mutually exclusive

^c^One incident involved injuries to both staff and a patient

#### Perpetrator categories

There were four main categories of perpetrators; perpetrators with (i) no medical problem, (ii) a physical health issue, (iii) an MBD, and (iv) repeat perpetrators.

#### No medical cause

Incidents associated with no medical cause most commonly occurred in the waiting room (33.3% *n* = 6) and on early shifts (38.9% *n* = 7). Five (27.8%) perpetrators were bystanders and 13 (72.7%) were patients who had no known medical reason to attend the ED or mild illness or injury that did not require treatment. The most common perceived causes of aggression were frustration with waiting and being denied a request. All incidents involved verbal violence (100% *n* = 18) and two (11.1%) involved physical violence. No injuries were caused by this cohort of perpetrators.

#### Physical health cause

Head injuries in conjunction with alcohol intoxication were the only physical health issues that occurred more than once. More than half (54.5% *n* = 6) were associated with an MBD. Four (36.4%) incidents involved verbal violence and five (45.5%) physical violence. One (9.1%) incident involved self-harm and four (36.4%) involved patients attempting to abscond. Staff were injured in two (18.2%) incidents.

#### Mental and behavioural disorders

MBDs were the most prevalent perpetrator category. This category was broken down into two main subgroups; PSU and MBDs other than PSU. These subgroups are not mutually exclusive, 25 (24.5%) cases associated with MBDs included both subgroups. An additional subgroup for complex MBDs has also been included.

#### Psychoactive substance use

Alcohol was the most common psychoactive substance reported (50.9% *n* = 27). Methamphetamine (37.7% *n* = 20) was the most common illicit drug followed by Cannabis (17.0% *n* = 9) and Heroin (3.8% *n* = 2). Six (11.3%) incidents involved multiple illicit substances and illicit substance use was reported but not specified in nine (17.0%) incidents. Young adults were the most frequent age group associated with PSU (50.9% *n* = 27). PSU was associated with 10 (37.0%) injuries. Ambulance transport occurred in 37 (69.8%) incidents involving PSU and police were involved in 34 (64.2%) incidents. Perpetrators were chemically sedated in 26 (49.1%) incidents and physically restrained in 16 (30.2%) which constitutes 50% of all perpetrators that were sedated and/or restrained. Incidents involving PSU most commonly occurred on night shifts (41.5% *n* = 22). Four (7.5%) patients stayed longer than 24 h in the ED and all were associated with PSU. Three (5.7%) were brought in with a police escort. The longest time spent in the ED was over 55 h for a patient awaiting admission to a psychiatric facility.

#### MBD other than PSU

**Fig. 1:**
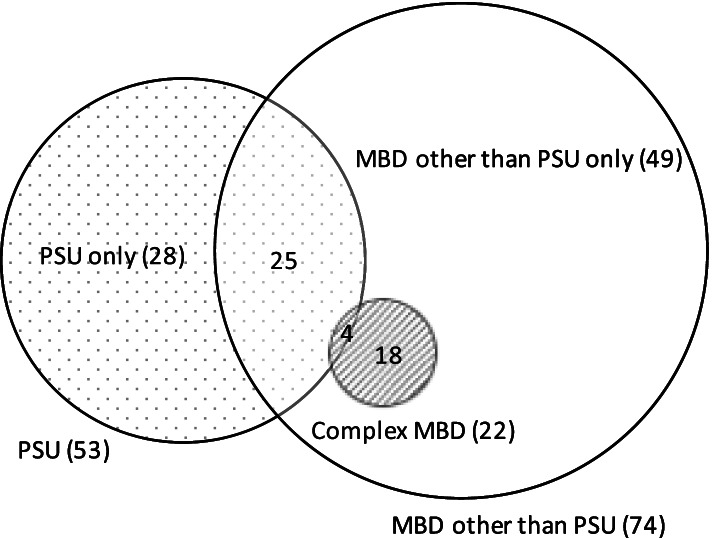
Categories of perpetrators with MBD

MBDs other than substance use included a variety of MBDs and is reflected in the MBD past history section of Table 2. It was often difficult to determine which MBD was of current concern as patient notes and incident reports frequently reported ‘mental health’ or ‘psychiatric’ issue instead of a specific MBD. This cohort was responsible for 18 (66.7%) injuries. Thirty two (43.2%) perpetrators were chemically sedated and 26 (35.1%) were restrained, which comprises the majority of perpetrators that were sedated (65.3%) and restrained (81.3%). Figure 1 displays the complex relationship between MBD subcategories including the complex MBD subcategory. Complex MBDs included dementia (31.8% *n* = 7), intellectual disability (27.3% *n* = 6), autism (18.2% *n* = 4), personality disorder (13.6% *n* = 3), and delirium (9.1% *n* = 2). Seven (31.8%) incidents in this category involved a perpetrator aged over 75 years. While 51.0% (52) of all MBD related incidents had police involvement, police were only involved in 22.7% (5) of complex MBD cases. In contrast to this, 59.1% (13) of these cases were transported by ambulance. Eleven (50.0%) perpetrators were sedated and eight (36.4%) were restrained. Security alerts for complex MBD patients most commonly occurred during late shifts (50% *n* = 11).

#### Repeat perpetrators

The 31 repeat perpetrators were predominantly male (66.7%) and either a young adult (43.3%) or middle aged (43.3%). They most commonly presented on late shifts (53.6%), were far more likely to become violent in the waiting room (46.2%) and were equally verbally (58.1%) and physically (58.1%) violent. The majority of incidents associated with repeat perpetrators were also associated with MBDs (93.5%). Twenty nine percent of repeat perpetrators were associated only with PSU, 41.9% were associated only with an MBD other than PSU and 22.6% involved both. Repeat perpetrators were associated with 16.1% injuries and 93.5% had a psychiatric past history.

## Discussion

### Main findings

The aim of this study was to identify the perpetrator and situational characteristics associated with security alerts in regional Australian emergency departments. This study provides much needed regional ED data and is the first study to apply ED data to the categories of perpetrators that ED staff differentiate between and approach differently.

This will assist with better understanding of the breadth and depth of the issue and for more targeted efforts to reduce violence. One notable finding was that mental and behavioural disorders (MBD) was a perpetrator characteristic present in the majority of security alerts. This characteristic was also associated with an increased severity of incidents and perpetrating multiple incidents of violence. Incidents most commonly occurred in the first 4 h following arrival at hospital, on late shifts and in ED cubicles. Almost all perpetrators were patients, most incidents involved verbal violence, and more than half involved physical violence. Many of the perpetrator and situational characteristics observed in this study are similar to previous studies, with the main differences being an increased rate of self-harm and attempted absconding and a higher incidence of perpetrators presenting with and having a past history of MBDs [1, 3, 12].

### Mental and behavioural disorders

One of the prominent outcomes of this study is the frequency and severity of violent incidents associated with MBDs in the emergency department. While similar studies did not report PSU as an MBD, this study found MBDs other than PSU remained the most prevalent perpetrator category and higher than other Australian studies [1, 9]. MBD presentations comprised 3.4% of all presentations to Victorian EDs in 2018 and 2019 [21] yet they are associated with 81.6% of the violent incidents in this study. Perpetrators with MBDs were associated with almost all of the injuries caused by violent behaviour in this study and MBDs other than PSU were associated with the majority of these injuries.

It has been argued that the ED is not an appropriate environment for many MBD patients, since MBD patients may have long wait times in an overly stimulating environment and often do not receive the care they require while in the ED [22–25]. The Australasian College for Emergency Medicine state that providing adequate community-based services for MBD patients should be a priority [23]. In the recent Covid-19 pandemic, two Australian studies have shown an increase in ED presentations for several MBDs in the context of decreasing total ED presentations [26, 27]. This may pose a unique challenge during times of crisis [23] and potentially lead to an increase in violence in the ED. In emergency situations where patients with MBDs do require the ED, Behavioural Assessment Units (BAU) have been recommended as a way to remove these patients from the chaotic ED environment [28, 29]. BAUs are co-located within the ED have been associated with decreased ED length of stay along with lower incidence of security alerts, mechanical restraint and therapeutic sedation, however controlled trials are still required to assess their effectiveness [28].

### Psychoactive substance use

PSU was associated with a large proportion of incidents in this study. The proportion is similar to both Australian and international studies, with an Australian study reporting up to 66% of incidents associated with PSU [1, 12]. Alcohol related presentations place a substantial burden on EDs [9]. Research is being conducted on the safety and effectiveness of diverting intoxicated patients away from ED to specialist services and this appears to be a promising approach to reduce PSU presentations to the ED [30].

### Repeat perpetrators

Repeat perpetrators are more challenging than other perpetrators and have a substantial impact on workplace violence in the ED [2, 7]. Studies from metropolitan EDs in Victoria have reported similar although slightly lower rates of incidents involving repeat perpetrators (< 20%) and called for research into interventions to reduce the impact of this group. ED nurses and paramedics have described a lack of consequences for the majority of violent repeat perpetrators and expressed their desire for violent repeat perpetrators to receive appropriate consequences. The consequences discussed were not always punitive and could involve including them in the follow-up of violent events [2, 7, 25].

### Multidisciplinary approach

A majority of incidents in this study involved multidisciplinary teams providing care for patients. Many patients arrived via ambulance and over half involved police. A similar study from regional Victoria showed a higher rate of 79.5% arrived via ambulance [3]. This is in contrast to the average rate of 26.0% for all patients arriving at Victorian EDs via ambulance [21]. Violence in the ED impacts multiple disciplines and likely requires a multidisciplinary approach to interventions; including ED staff, ambulance services, police, psychiatric and community mental health services. The diversion of patients with MBD and PSU into community-based services is one example of a multidisciplinary approach [22, 24, 25, 30]. Violence in the ED appears to be part of a larger, societal problem with violence and risk-taking behaviour [2, 6, 9], and public health approaches are thus needed to address the societal component of violence in the ED. Local health department engagement with public health projects has been associated with reduced likelihood of ED presentations involving PSU as well as preventing PSU disorders and reducing violence and crime rates in the community [31].

### Strengths and limitations

This is the first study to audit incident reports and patient medical records associated with security alerts in regional EDs. Accessing patient medical records was a strength of this study, validating the information found in incident reports and providing patient characteristics that were inaccessible from incident reports. One key limitation of this study is that the extent of under-reporting of violent incidents is not known. The incidence of violent incidents was lower at site 1, which may indicate different reporting procedures between sites. Missing data for many of the variables and the assumption that it was missing completely at random may limit the results. The study relies on the accuracy of documentation in the incident reports and patient notes. The documentation of MBDs was not standardised and often did not report a specific MBD, this will likely result in the Complex MBD category being underrepresented in the results. In addition, there was a lack of blood alcohol and illicit substance screening recorded in the patient notes; rather, history of substance abuse was often based on self-report. A prospective study design would likely allow for specific criteria to be recorded.

## Conclusion

Violence incidents in the ED are often complex, patients present with multiple issues and are managed across disciplines. Interventions need to extend from one size fits all approaches to targeting specific perpetrator groups. MBDs were the most frequent patient factors present in security alerts and were associated with increased severity of incidents. Public health and multidisciplinary approaches focussing on MBDs are needed to reduce workplace violence in EDs and improve care for patients with MBDs.

## Data Availability

The full dataset is available via Figshare (10.6084/m9.figshare.14907861.v1).

## Abbreviations

ED: Emergency department
MBD: Mental and behavioural disorders
PSU: Psychoactive substance use
BAU: Behavioural Assessment Unit
